# Supplementary material for: Climate-sensitive zoonotic diseases transmissible by companion animals: A scoping review protocol
Source: PLoS One. 2025 Jun 10;20(6):e0325568. doi: 10.1371/journal.pone.0325568 (PMC12151349; doi:10.1371/journal.pone.0325568)
Supplement: S1 File — (DOCX) [file pone.0325568.s001.docx]

**Appendix I:** Proposed keywords and controlled vocabulary for the key concepts of meteorological factors, companion animals, human health, and companion animal zoonotic diseases.

| **Key concepts** | **Keywords** | **MeSH headings** |
| --- | --- | --- |
| Meteorological factors | Climat* OR Meteorological OR Weather OR Season* OR Temperature? OR Precipitation OR Rain* OR Humid* OR Wind OR Ultraviolet radiation OR Solar radiation OR Wildfire? OR Flood? OR Drought? OR Heatwave? OR Heat wave? | EXP Climate change  EXP Weather |
| Companion animals  (cats and dogs) | Cat OR Cats OR  Dog OR Dogs OR  Companion animal? OR  Feline OR Canine OR Felis catus OR Canis familiaris OR Canis lupus familiaris | Cats  Dogs |
| Human health  (individual-level) | Human? OR People? OR Person? OR Individual? OR Patient? OR Public health | Humans |
| Human health  (population-level) | Population-level OR Area-level OR City-level OR State-level OR Provincial-level OR Region* OR County OR Counties OR Neighbourhood? OR Neighborhood? OR Municipalit* OR District OR Ecological |  |
| **Parasite** | | |
| Ascariasis  (*Ascaris lumbricoides*) | Ascaris lumbricoides OR  Ascariasis | Ascaris lumbricoides  Ascariasis |
| Raccoon roundworm  (*Baylisacaris procyonis*) | Baylisacaris procyonis OR  Ascaridida OR  Raccoon roundworm | EXP Ascaridida infections |
| Cheyletiellosis  (*Cheyletiella* spp.) | Cheyletiell* OR  Walking dandruff |  |
| Cryptosporidiosis  (*Cryptosporidium* spp.) | Cryptosporidi* | EXP Cryptosporidium  Cryptosporidiosis |
| Flea tapeworm  (*Dipylidium caninum*) | Dipylidium caninum OR  Common flea tapeworm |  |
| Dirofilariasis  (*Dirofilaria immitis*) | Dirofilaria immitis OR  Dirofilariasis OR  Heartworm | Dirofilaria immitis  Dirofilariasis |
| Echinococcosis  (*Echinococcus* spp.) | Echinococc* OR  Hydatid* OR  Alveolar cyst | EXP Echinococcus  EXP Echinococcosis |
| *Eucoleus aerophilus* | Eucoleus aerophilus OR  Lungworm |  |
| *Ctenocephalides* spp. | Ctenocephalides felis OR  Ctenocephalides canis OR  Cat flea OR  Dog flea | Ctenocephalides |
| Giardiasis  (*Giardia* spp.) | Giardia* OR  Lamblia* | EXP Giardia  Giardiasis |
| Ancylostomiasis  (*Ancylostoma* spp.) | Ancylostom* OR  Hookworm | Ancylostoma  Ancylostomiasis |
| Leishmaniasis  (*Leishmania* spp.) | Leishmania* | EXP Leishmania  EXP Leishmaniasis |
| Notoederic mange  (*Notoedres cati*) | Notoedres cati OR  Mange |  |
| Rat mite  (*Ornithonyssus bacoti*) | Ornithonyssus bacoti OR  Rat mite |  |
| Ear mite  (*Otodects cynotis*) | Otodects cynotis OR  Ear mite |  |
| Scabies  (*Sarcoptes scabiei*) | Sarcoptes scabiei OR  Sarcoptic mange OR  Scabies | Sarcoptes scabiei  Scabies |
| Strongyloidiasis  (*Strongyloides stercoralis*) | Strongyloides stercoralis OR  Threadworm OR  Strongyloidiasis | Strongyloides stercoralis  Strongyloidiasis |
| Taeniasis  (*Taenia* spp.) | Taenia* | EXP Taenia  EXP Taeniasis |
| Toxascaris  (*Toxocara* spp.) and  Toxascariasis  (*Toxascaris* spp.) | Toxocar* OR  Toxascari* | EXP Toxocara  Toxascaris  EXP Toxocariasis  Toxascariasis |
| Toxoplasmosis  (*Toxoplasma gondii*) | Toxoplasma gondii OR  Toxoplasmosis | EXP Toxoplasma  EXP Toxoplasmosis |
| Trichuriasis  (*Trichuris vulpis*) | Trichuris vulpis OR  Trichuriasis OR  Trichocephaliasis OR  Whipworm | Trichuris  Trichuriasis |
| *Trichomonas foetus* | Trichomonas foetus | Trichomonas foetus |
| Trypanosomiasis  (*Trypanosoma cruzi*) | Trypanosoma cruzi OR  Trypanosomiasis OR  Chaga’s disease OR  Chagas disease | Trypanosoma cruzi  EXP Chagas disease  EXP Trypanosomiasis |
| **Bacteria** | | |
| *Anaerobiospirillum* spp. | Anaerobiospirillum | Anaerobiospirillum |
| Anaplasmosis  (*Anaplasma Phagocytophilum*) | Anaplasma phagocytophilum OR  Anaplasm* OR  Granulocytic ehrlichiosi | Anaplasma phagocytophilum  Anaplasmosis  EXP Ehrlichiosis |
| *Arcobacter butzleri* | Arcobacter butzleri | Arcobacter |
| Anthrax  (*Bacillus anthracis*) | Bacillus anthracis OR  Anthrax | Bacillus anthracis  Anthrax |
| Cat scratch disease  (*Bartonella henselae*) | Bartonella henselae OR  Rochalimaea henselae OR  Cat scratch disease OR  Cat scratch fever OR  Epithelioid angiomatosis OR  Bacillary angiomatosis OR  Bacillary peliosis | Bartonella henselae  Angiomatosis, bacillary  Cat-Scratch disease |
| *Bartonella clarridgeiae* | Bartonella clarridgeiae |  |
| Lyme disease  *(Borrelia burgdorferi)* | Borrelia Burgdorferi OR  Lyme* | Borrelia burgdorferi  EXP Lyme disease |
| Kennel cough  (*Bordetella bronchiseptica*) | Bordetella bronchiseptica OR  Kennel Cough | Bordetella bronchiseptica |
| *Bergeyella zoohelcum* | Bergeyella zoohelcum |  |
| Brucellosis  (*Brucella canis*) | Brucella canis OR  Brucellosis OR  Cyprus fever OR  Gibraltar fever OR  Malta fever OR  Rock fever OR  Undulant fever | Brucella canis  EXP Brucellosis |
| Campylobacteriosis  (*Campylobacter* spp.) | Campylobacter* | EXP Campylobacter  Campylobacter infections |
| *Capnocytophaga* spp. | Capnocytophaga | Capnocytophaga |
| *Chlamydophila felis* | Chlamydophila felis |  |
| Psittacosis  (*Chlamydophila psittaci*) | Chlamydophila psittaci OR  Chlamydia psittaci OR  Parrot fever OR  Psittacosis OR  Ornithosis | Chlamydophila psittaci  Psittacosis |
| *Clostridium difficile* | Clostridium difficile OR Clostridioides difficile | Clostridioides difficile  EXP Clostridium infections |
| *Clostridium perfringens* | Clostridium perfringens OR  Clostridioides perfringens OR  Clostridium welchii | Clostridium pefringens  EXP Clostridium infections |
| *Corynebacterium ulcerans* | Corynebacterium ulcerans |  |
| Q fever  (*Coxiella burnetii*) | Coxiella burnetii OR  Q fever OR  Query fever | Coxiella burnetii  Q fever |
| *Edwardsiella tarda* | Edwardsiella tarda OR  Edwardsiella anguillimortifera | Edwardsiella tarda |
| Ehrlichiosis  (*Ehrlichia* spp.) | Ehrlichia canis OR  Ehrlichia chaffeensis OR  Ehrlichia ewingii OR  Ehrlichiosis | Ehrlichia canis  Ehrlichia chaffeensis  EXP Ehrlichiosis |
| *Eikenella corrodens* | Eikenella corrodens OR  Bacteroides corrodens OR  Ristella corrodens | Eikenella corrodens |
| *Enterococcus* spp. | Enterococcus faec* OR  Streptococcus faec* OR  (Group D ADJ2 Streptococcus) | Enterococcus faecium  Enterococcus faecalis |
| *Escherichia coli* O157 | Escherichia coli O157 OR  E coli O157 OR  VTEC OR  Shiga toxin* OR  Vero toxin? | Escherichia coli O157  EXP Escherichia coli infections  EXP Shiga toxins |
| Tularemia  (*Francisella tularensis*) | Francisella tularens* OR  Bacterium tularensis OR  Brucella tularensis OR  Pasteurella tularensis OR  Tularemia | Francisella tularensis  Tularemia |
| *Helicobacter* spp. | Helicobacter | EXP Helicobacter  Helicobacter infections |
| Leptospirosis  (*Leptospira*spp.) | Leptospir* OR  Cane Cutter fever OR  Canicola fever OR  Mud fever OR  Rice Field fever OR  Stuttgart disease OR  Swineherd’s disease OR  Weil disease OR  Weil’s disease | EXP Leptospira  EXP Leptospirosis |
| Listeriosis  (*Listeria monocytogenes*) | Listeria monocytogenes OR  Listeriosis | Listeria monocytogenes  EXP Listeriosis |
| Bovine tuberculosis  (*Mycobacterium* *tuberculosis*) | Mycobacterium bovis OR  Tuberculosis OR  Koch disease OR  Koch’s disease | Mycobacterium bovis  Tuberculosis, Bovine  Mycobacterium tuberculosis  EXP Tuberculosis |
| Pasteurellosis  (*Pasteurella multocida*) | Pasteurella multocida OR  Pasteurellosis OR  Shipping fever OR  Haemorrhagic septicemia | Pasteurella multocida  Pasteurellosis, Pneuomonic  Hemorrhagic septicemia |
| *Plesiomonas shigelloides* | Plesiomonas shigelloides | Plesiomonas |
| Rat bite fever  (*Streptobacillus moniliformis* and S*pirillum minus*) | Streptobacillus moniliformis OR  Spirillum minus OR  Rat Bite fever OR  Haverhill fever | Rat-bite fever |
| Rickettsiosis  (*Rickettsia felis*) | Rickettsia felis OR  Flea-borne spotted fever OR  Rickettsiosis | Rickettsia felis  EXP Rickettsia infections |
| Rocky mountain spotted fever  (*Rickettsia rickettsii*) | Rickettsia rickettsii OR  Rocky mountain spotted fever OR  Brazilian spotted fever OR  Sao Paulo Typhus | Rickettsia rickettsii  Rocky mountain spotted fever |
| Salmonellosis  (*Salmonella enterica*) | Salmonella enterica OR  Salmonellosis | EXP Salmonella enterica  EXP Salmonella infections |
| *Staphylococcus* spp. | Staphylococcus aureus OR  Staphylococcus  pseudintermedius OR  Staphylococcus intermedius OR Staphylococcus schleiferi | EXP Staphylococcus aureus  Staphylococcus intermedius  EXP Staphylococcal infections |
| *Streptococcus* spp. | Streptococcus canis OR  (Group A ADJ2 Streptococcus) OR  Streptococcus pyogenes OR  Scarlet fever OR  Rheumatic fever OR  Rheumatic arthritis OR  Rheumatism | Streptococcus pyogenes  Scarlet fever  EXP Rheumatic fever |
| Yersiniosis  (*Yersinia enterocolitica*) | Yersinia enterocolitica OR  Yersiniosis | Yersinia enterocolitica  EXP Yersinia infections |
| Plague  (*Yersinia pestis*) | Yersinia pestis OR  Plague OR  Black death | Yersinia pestis  Plague |
| Far east scarlet-like fever  *(Yersinia pseudotuberculosis*) | Yersinia pseudotuberculosis OR  Far east scarlet-like fever | Yersinia pseudotuberculosis  Yersinia pseudotuberculosis infections |
| **Virus** | | |
| Cowpox  (Cowpox virus) | Cowpox OR  Cow pox | Cowpox virus  Cowpox |
| European bat lyssavirus  (EBLV-1 and EBLV-2) | European bat lyssavirus OR  EBLV-1 OR  EBLV-2 |  |
| Hantavirus  (Sin Nombre virus) | Hantavirus OR  Sin Nombre virus OR  Four Corners virus OR  Muerto Canyon virus | Sin Nombre virus  Hantavirus pulmonary syndrome |
| Herpes simplex virus (HSV) | Herpes simplex virus OR  Herpesvirus | EXP Simplexvirus  EXP Herpes simplex |
| Avian influenza  (H1N1 and H5N1) | Influenza A virus OR  Influenza virus* type A OR  H5N1 OR  H1N1 OR  Avian flu OR  Avian influenza OR  Fowl plague | Influenza A Virus, H5N1 subtype  Influenza A Virus, H1N1 subtype  Influenza in Birds |
| Lymphocytic choriomeningitis (LCM virus) | Lymphocytic choriomeningitis OR  LCM virus* OR  LCMV OR  Armstrong syndrome | Lymphocytic choriomeningitis virus  Lymphocytic choriomeningitis |
| Monkeypox  (MPOX virus) | Monkeypox OR  Monkey pox OR  MPOX | Monkeypox virus  Monkeypox |
| Nipah virus | Nipah virus OR  Nipah henipavirus | Nipah virus  Henipavirus infections |
| Rabies  (Rabies virus) | Rabies | Rabies virus  Rabies |
| **Fungi** | | |
| Aspergillosis  (*Aspergillus fumigatus*) | Aspergillus fumigat* OR  Aspergillosis | Aspergillus fumigatus  EXP Aspergillosis |
| Blastomycosis  (*Blastomyces* spp.) | Blastomyces dermatitidis OR Blastomyces brasiliensis OR Blastomycosis OR  Gilchrist disease OR  Gilchrist's disease OR  Paracoccidioid* | Blastomyces  Blastomycosis  Paracoccidioides  Paracoccidioidomycosis |
| Coccidioidomycosis  (*Coccidioides* spp.) | Coccidioides immitis OR  Coccidioides posadasii OR  Coccidioidomycosis OR  Valley fever | Coccidioides  Coccidioidomycosis |
| Cryptococcosis  (*Cryptococcus* spp.) | Cryptococc* OR  Torulosis | EXP Cryptococcus  EXP Cryptococcosis |
| Dermatophytosis  (*Dermatophytes*) | Dermatophyt* OR  Epidermophytos* OR  Ringworm OR  Trichophyto* OR  Tinea corporis | EXP Tinea |
| Encephalitozoonosis  (*Encephalitozoon* spp.) | Encephalitozoon cuniculi OR Encephalitozoon hellem OR  Encephalitozoonosis | Encephalitozoon cuniculi  Encephalitozoonosis |
| Microsporidiosis  (*Enterocytozoon bieneusi*) | Enterocytozoon bieneusi OR  Microsporidiosis | Enterocytozoon  EXP Microsporidiosis |
| Histoplasmosis  (*Histoplasma* spp.) | Histoplasma capsulatum OR  Histoplasma duboisii OR  Histoplasmosis | Histoplasma  Histoplasmosis |
| *Malassezia pachydermatis* | Malassezia pachydermatis |  |
| Sporotrichosis  (*Sporothrix schenckii*) | Sporothrix schenckii OR  Sporotrichosis | Sporothrix  Sporotrichosis |
