# Supplementary material for: Climate-sensitive zoonotic diseases transmissible by companion animals: A scoping review protocol
Source: PLoS One. 2025 Jun 10;20(6):e0325568. doi: 10.1371/journal.pone.0325568 (PMC12151349; doi:10.1371/journal.pone.0325568)
Supplement: S2 File — (DOCX) [file pone.0325568.s002.docx]

**Appendix II**: Search terms for MEDLINE via OVID, executed February 1, 2024

| **No.** | **Concept** | **Search String** |
| --- | --- | --- |
| 1 | Meteorological factors | (exp Climate change/ or exp Weather/) or (Climat* or Meteorological or Weather or Season* or Temperature? or Precipitation or Rain* or Humid* or Wind or Ultraviolet radiation or Solar radiation or Wildfire? or Flood* or Drought? or Heatwave? or Heat wave?).ab.kw.ti |
| 2 | Companion animals | (Cat or Cats or Dog or Dogs or Companion animal or Feline or Canine or Felis catus or Canis familiaris or Canis lupus familiaris).ab,ti,kw. |
| 3 | Human  (individual-level) | (Humans/) or (Human? or People? or Person? or Individual? or Patient? or Public health).ab,kw,ti. |
| 4 | Human  (population-level) | (Population-level or Area-level or City-level or State-level or Provincial-level or Region* or County or Counties or Neighbourhood? or Neighborhood? or Municipalit* or District) ab.kw.ti |
| 5 | Zoonoses | (Zoonotic disease* or zoonotic pathogen* or zoonoses or zoonosis or zoonotic).ab,kw,ti. |
| 6 | Parasite | (Ascaris lumbricoides/ or Ascariasis/ or exp Ascaridida infections/ or exp Cryptosporidium/ or Cryptosporidiosis/ or Dirofilaria immitis/ or Dirofilariasis/ or exp Echinococcus/ or exp Echinococcosis/ or Ctenocephalides/ or exp Giardia/ or Giardiasis/ or Ancylostoma/ or Ancylostomiasis/ or exp Leishmania/ or exp Leishmaniasis/ or Sarcoptes scabiei/ or Scabies/ or Strongyloides stercoralis/ or Strongyloidiasis/ or exp Taenia/ or exp Taeniasis/ or exp Toxocara/ or Toxascaris/ or exp Toxocariasis/ or Toxascariasis/ or exp Toxoplasma/ or exp Toxoplasmosis/ or Trichuris/ or Trichuriasis/ or Trichomonas foetus/ or Trypanosoma cruzi/ or exp Chagas disease/ or exp Trypanosomiasis/) or (Ascaris lumbricoides or Ascariasis or Baylisacaris procyonis or Ascaridida or Raccoon roundworm or Cheyletiell* or Walking dandruff or Cryptosporidi* or Dipylidium caninum or Common flea tapeworm or Dirofilaria immitis or Dirofilariasis or Heartworm or Echinococc* or Hydatid* or Alveolar cyst or Eucoleus aerophilus or Lungworm or Ctenocephalides felis or Ctenocephalides canis or Cat flea or Dog flea or Giardia* or Lamblia* or Ancylostom* or Hookworm or Leishmania* or Notoedres cati or Mange or Ornithonyssus bacoti or Rat mite or Otodects cynotis or Ear mite or Sarcoptes scabiei or Sarcoptic mange or Scabies or Strongyloides stercoralis or Threadworm or Strongyloidiasis or Taenia* or Toxocar* or Toxascari* or Trichuris vulpis or Trichuriasis or Trichocephaliasis or Whipworm or Trypanosoma cruzi or Trypanosomiasis or Chaga's disease or Chagas disease).ab,kw,ti. |
| 7 | Bacteria | (Anaerobiospirillum/ or Anaplasma phagocytophilum/ or Anaplasmosis/ or exp Ehrlichiosis/ or Arcobacter/ or Bacillus anthracis/ or Anthrax/ or Bartonella heneselae/ or Angiomatosis, Bacillary/ or Cat-Scratch Disease/ or Borrelia burgdorferi/ or exp Lyme Disease/ or Bordetella bronchiseptica/ or Brucella canis/ or exp Brucellosis/ or exp Campylobacter/ or Campylobacter infections/ or Capnocytophaga/ or Chlamydophila psittaci/ or Psittacosis/ or Clostridioides difficile/ or exp Clostridium infections/ or Clostridium pefringens/ or exp Clostridium infections/ or Coxiella burnetii/ or Q fever/ or Edwardsiella tarda/ or Ehrlichia canis/ or Ehrlichia chaffeensis/ or exp Ehrlichiosis/ or Eikenella corrodens/ or Enterococcus faecium/ or Enterococcus faecalis/ or Escherichia coli O157/ or exp Escherichia coli infections/ or exp Shiga toxins/ or Francisella tularensis/ or Tularemia/ or exp Helicobacter/ or Helicobacter infections/ or exp Leptospira/ or exp Leptospirosis/ or Listeria monocytogenes/ or exp Listeriosis/ or Mycobacterium bovis/ or Tuberculosis, Bovine/ or Mycobacterium tuberculosis/ or exp Tuberculosis/ or Pasteurella multocida/ or Pasteurellosis, Pneuomonic/ or Hemorrhagic septicemia/ or Plesiomonas/ or Rat-bite fever/ or Rickettsia felis/ or exp Rickettsia infections/ or Rickettsia rickettsii/ or Rocky mountain spotted fever/ or exp Salmonella enterica/ or exp Salmonella infections/ or exp Staphylococcus aureus/ or Staphylococcus intermedius/ or exp Staphylococcal infections/ or Streptococcus pyogenes/ or Scarlet fever/ or exp Rheumatic fever/ or Yersinia enterocolitica/ or exp Yersinia infections/ or Yersinia pestis/ or Plague/ or Yersinia pseudotuberculosis/ or Yersinia pseudotuberculosis infections/) or (Anaerobiospirillum or Anaplasma phagocytophilum or Anaplasm* or Granulocytic ehrlichiosis or Arcobacter butzleri or Bacillus anthracis or Anthrax or Bartonella henselae or Rochalimaea henselae or Cat Scratch disease or Cat Scratch fever or Epithelioid Angiomatosis or Bacillary Angiomatosis or Bacillary Peliosis or Bartonella clarridgeiae or Borrelia Burgdorferi or Lyme* or Bordetella bronchiseptica or Kennel cough or Bergeyella zoohelcum or Brucella canis or Brucellosis or Cyprus fever or Gibraltar fever or Malta fever or Rock fever or Undulant fever or Campylobacter* or Capnocytophaga or Chlamydophila felis or Chlamydophila psittaci or Chlamydia psittaci or Parrot fever or Psittacosis or Ornithosis or Clostridium difficile or Clostridioides difficile or Clostridium perfringens or Clostridioides perfringens or Clostridium welchii or Corynebacterium ulcerans or Coxiella burnetii or Q fever or Query fever or Edwardsiella tarda or Edwardsiella anguillimortifera or Ehrlichia canis or Ehrlichia chaffeensis or Ehrlichia ewingii or Ehrlichiosis or Eikenella corrodens or Bacteroides corrodens or Ristella corrodens or Enterococcus faec* or Streptococcus faec* or (Group D adj2 Streptococcus) or Escherichia coli O157 or E coli O157 or VTEC or Shiga toxin* or Vero toxin? or Francisella tularens* or Bacterium tularensis or Brucella tularensis or Pasteurella tularensis or Tularemia or Helicobacter or Leptospir* or Cane Cutter fever or Canicola fever or Mud fever or Rice Field fever or Stuttgart disease or Swineherd's disease or Weil disease or Weil's disease or Listeria monocytogenes or Listeriosis or Mycobacterium bovis or Tuberculosis or Koch disease or Koch's disease or Pasteurella multocida or Pasteurellosis or Shipping fever or Haemorrhagic septicemia or Plesiomonas shigelloides or Streptobacillus moniliformis or Spirillum minus or Rat Bite fever or Haverhill fever or Rickettsia felis or Flea-borne spotted fever or Rickettsiosis or Rickettsia rickettsii or Rocky mountain spotted fever or Brazilian spotted fever or Sao Paulo Typhus or Salmonella enterica or Salmonellosis or Staphylococcus aureus or Staphylococcus pseudintermedius or Staphylococcus intermedius or Staphylococcus schleiferi or Streptococcus canis or (Group A adj2 Streptococcus) or Streptococcus pyogenes or Scarlet fever or Rheumatic fever or Rheumatic arthritis or Rheumatism or Yersinia enterocolitica or Yersiniosis or Yersinia pestis or Plague or Black death or Yersinia pseudotuberculosis or Far east scarlet-like fever).ab,kw,ti. |
| 8 | Virus | (Cowpox virus/ or Cowpox/ or Sin Nombre virus/ or Hantavirus pulmonary syndrome/ or exp Simplexvirus/ or exp Herpes simplex/ or Influenza A Virus, H5N1 subtype/ or Influenza A Virus, H1N1 subtype/ or Influenza in Birds/ or Lymphocytic choriomeningitis virus/ or Lymphocytic choriomeningitis/ or Monkeypox virus/ or Monkeypox/ or Nipah Virus/ or Henipavirus infections/ or Rabies virus/ or Rabies/) or (Cowpox or Cow pox or European bat lyssavirus or EBLV-1 or EBLV-2 or Hantavirus or Sin Nombre virus or Four Corners virus or Muerto Canyon virus or Herpes simplex virus or Herpesvirus or Influenza A virus or Influenza virus* type A or H5N1 or H1N1 or Avian flu or Avian influenza or Fowl plague or Lymphocytic choriomeningitis or LCM virus* or LCMV or Armstrong syndrome or Monkeypox or Monkey pox or MPOX or Nipah virus or Nipah henipavirus or Rabies).ab,kw,ti. |
| 9 | Fungi | (Aspergillus fumigatus/ or exp Aspergillosis/ or Blastomyces/ or Blastomycosis/ or Paracoccidioides/ or Paracoccidioidomycosis/ or Coccidioides/ or Coccidioidomycosis/ or exp Cryptococcus/ or exp Cryptococcosis/ or exp Tinea/ or Encephalitozoon cuniculi/ or Encephalitozoonosis/ or Enterocytozoon/ or exp Microsporidiosis/ or Histoplasma/ or Histoplasmosis/ or Sporothrix/ or Sporotrichosis/) or (Aspergillus fumigat* or Aspergillosis or Blastomyces dermatitidis or Blastomyces brasiliensis or Blastomycosis or Gilchrist disease or Gilchrist's disease or Paracoccidioid* or Coccidioides immitis or Coccidioides posadasii or Coccidioidomycosis or Valley fever or Cryptococc* or Torulosis or Dermatophyt* or Epidermophytos* or Ringworm or Trichophyto* or Tinea corporis or Encephalitozoon cuniculi or Encephalitozoon hellem or Encephalitozoonosis or Enterocytozoon bieneusi or Microsporidiosis or Histoplasma capsulatum or Histoplasma duboisii or Histoplasmosis or Malassezia pachydermatis or Sporothrix schenckii or Sporotrichosis).ab,kw,ti. |
| 10 |  | 3 or 4 |
| 11 |  | 6 or 7 or 8 or 9 |
| 12 | Review #1 | 1 and 2 and (5 or 11) |
| 13 | Review #2 | 1 and 10 and 11 |
