# Supplementary material for: Climate-sensitive zoonotic diseases transmissible by companion animals: A scoping review protocol
Source: PLoS One. 2025 Jun 10;20(6):e0325568. doi: 10.1371/journal.pone.0325568 (PMC12151349; doi:10.1371/journal.pone.0325568)
Supplement: S3 File — (DOCX) [file pone.0325568.s003.docx]

**Appendix III**: Proposed data extraction form

| **Study characteristics** | |
| --- | --- |
| Author(s) |  |
| Year of Publication |  |
| Study location |  |
| Study objectives |  |
| Study design (Experimental or Observational) |  |
| Sampling unit (Individual or Population level) |  |
| Sample size |  |
| What species were included? |  |
| What analytical methods were used? |  |
| What companion animal zoonosis was evaluated? |  |
| How was the outcome measured? |  |
| What climatic factor(s) were evaluated? |  |
| How was the exposure measured? |  |
| **Evidence for climate sensitivity** | |
| Objective #2*: Were climatic risk factors described using a measure of association or effect? If yes, provide details. |  |
| Objective #3: Were spatial patterns and/or seasonality of disease incidence described in the context of climate change. If yes, provide details. |  |
| Objective #4**: Were projected impacts of climate change on the risk of zoonotic diseases included? If yes, provide details. |  |
| **Study limitations** | |
| What study limitations were reported by the author(s)? |  |
| What study limitations can be found by the reviewer? |  |
| **Evidence for zoonotic transmission** | |
| Was zoonotic transmission discussed? If yes, please report the transmission route and species involved. |  |

*Include confounders controlled, statistical significance, and statistical measures.

**For example, projected impacts of specified climate emission scenarios on disease incidence.
